# Pictures versus words: can we use a pictorial scale to measure child health-related quality of life?

**DOI:** 10.3389/fpubh.2024.1398944

**Published:** 2024-07-29

**Authors:** Tracy Chor Wai Tang, Mun Wong, Jian-Bin Li, Derwin King Chung Chan

**Affiliations:** The Education University of Hong Kong, Tai Po, Hong Kong SAR, China

**Keywords:** pictorial scales, health-related quality of life, wellbeing, child, pictorial scale development

## Abstract

Measuring health-related quality of life (HRQoL) is important because it can serve as an indicator or a predictor of subsequent mortality or morbidity. HRQoL has been shown to be directly related to child growth and development and indirectly related to the healthcare costs of young children. Existing measures of HRQoL in children have heavily relied on traditional questionnaires that use age-suited versions or parent proxy questionnaires. However, both of these methods may present with different types of biases and may misrepresent underlying HRQoL. The current mini reivew will first illustrate these methodological limitations and highlight the potential use of pictorial scales in addition to discussing their suitability for specifically measuring HRQoL as an alternative. We will also synthesize existing recommendations on the development of pictorial scales to provide a protocol as a recommendation to researchers who are aiming to develop an overall HRQoL pictorial scale that is suited for children.

## Introduction

Health-related quality of life (HRQoL) refers to one’s general health satisfaction and functioning (1–9). Specifically, it encompasses multidimensional aspects of overall wellbeing and includes physical health, emotional health, social health and school/work functioning, all of which may be impacted by individuals’ health status. HRQoL is particularly important for reliable measurement in pediatric populations because current well-being can affect a wide variety of detrimental outcomes, such as morbidity, mortality, healthcare utilization/costs and parental stress (10–12). In this paper, we will first discuss the challenges and limitations of measuring child HRQoL using traditional questionnaire methods. We will then discuss the potential of a pictorial scale for addressing some of the methodological challenges and limitations in measuring child HRQoL using traditional methods. Finally, we will synthesize existing theoretical methods and recommend a three-phase approach for developing a pictorial HRQoL.

### Challenges and limitations of parent proxy measures of child HRQoL

Measures of overall child HRQoL, such as the Child Health Questionnaire (13), have traditionally relied on self-reported questionnaires completed by parent-proxy, where parents are given a series of statements and are asked to rate their child’s health on their behalf. However, studies comparing parent proxy reports and child self-reported formats have revealed a significant disparity between the two, where some parents overestimate or underestimate their child health-related quality of life (3, 5, 14, 15). Subsequent discussions suggested that it may occur because parents use a different “lens” to evaluate their child’s HRQoL. As such, parent perceptions may differ from those of their children. Qualitative investigations have also shown that parents may have a greater ability to recall a child’s externalization of a problem, such as being aggressive, crying, or screaming (physical aspect), as it is more observable (3, 16, 17). On the other hand, emotional problems, such as sadness, anxiety and worry (emotional aspects), are less observable phenomena, making it harder for parents to notice and remember them. These forms of recall or perception bias of parents subsequently raise concerns about whether the estimates of child HRQoL by parent-proxy measures misrepresent child HRQoL. The results of such further stimulated researchers to derive age-suited versions of questionnaires where researchers aimed to use age-suitable language to measure self-reported HRQoL in children.

### Age-suited questionnaire versions for measuring child HRQoL

There are many age-suited questionnaires currently available in the literature, such as the KIDSCREEN (18). However, one of the most well-known HRQoL scales was developed by Varni et al. (8) called the Pediatric Quality of Life Inventory (PedsQL). This questionnaire has both parent proxy (aged 2+) and child self-reported versions (aged 5+). Although there are versions suitable for younger children, it has been argued that completing traditional questionnaires could still be cognitively challenging for children (19–22). These challenges include the length and duration needed to complete the questionnaire, children’s ability to grasp complex/abstract constructs, children’s ability to relate these questions to their daily life and children’s developmental literacy (14, 22–27, 28, see Table 1).

**Table 1:** Comparison of traditional questionnaires versus pictorial questionnaires.

Questionnaire type	Format of item	Response style	Distribution medium	Visual representation	Accessibility	Abstract concepts	Cognitive load
Traditional questionnaire	Words	Numeric Scale Yes/No	Online/Paper	Participant own visualization	Proxy measures available	Participants own visualization	Read, understand, relate to question (literacy)
Pictorial questionnaire	Pictures	Picture Likert Scale Dichotomous pictures	Online/Paper	Representative scenario visualization	Accessible to individuals with disabilities or impairments	Visual representation aid	Convey items with visual examples resulting in less cognitive load

In addition to these factors, asking children to read a long list of written items in traditional questionnaires can reduce their interest, motivation and concentration in completing the questionnaires to their best ability. As such, the loss of interest or motivation may interfere with the completion of the latter items by other forms of response bias, such as acquiescence or recency bias (23, 24), which impairs the accuracy of the measures. Children with developmental delay, attention deficit, dyslexia, other special needs, or other health conditions in a medical setting are likely to find such questionnaires even more challenging as they may be affected by fatigue or their condition resulting in lower cognitive functioning/attention span (25–27). It is therefore important to address these biases and barriers, as the literature/research often compares diseased/recovering individuals to healthy individuals for a direct comparison of HRQoL as an indicator of overall health. Given the methodological limitations and barriers in using traditional HRQoL measures, we argue that a pictorial scale may be a plausible solution.

### What is a pictorial scale and why?

Pictorial scales present questionnaire items using static pictures instead of literary items, eliminating the language barrier of traditional questionnaires (20–22, 28–33). Pictorial scales use image-based elements to express the meaning of a question (item), where participants are typically presented with pictures and asked how relatable the specific situation, scenario, behavior or psychological status is to them. In accompanying the pictorial item, a pictorial Likert scale could also be used as a response style for participants to rate their differing degrees of relatability or agreeableness. For example, a Smiley face Likert scale (34) can have varying degrees of sad/neutral/happy faces, indicating the degree of relatableness/agreement, of which participants’ responses should be synonymous with those of a Likert scale in traditional questionnaires. With the interface much like a children’s book, pictorial scales can help children overcome the cognitive barrier of questionnaire completion by lowering the difficulty level, which means that children are more likely to be able to complete questionnaires on their own with minimal guidance or interference from parents or research personnel. It has also been suggested that pictorial scales can break the age barrier of questionnaires by allowing children younger than 5 years of age to complete the survey items, making the assessment tool more child friendly, more enjoyable, and less cognitively demanding (19, 21).

A summary review published by Sauer et al. (29) identified 57 studies that developed pictorial scales for different research topics. Most of these scales for children measure complex concepts, such as personality, family aggression, healthy diet, and anxiety, and they often apply relatable scenarios as a medium (22, 28, 33, 35). More importantly, some of these pictorial scales have been validated statistically and shown to be comparable to those of the corresponding self-reported measures or even objective measures (20, 31–33, 36). For example, a pictorial scale of perceived water competence in young children not only established content and face validity during scale development but was also successfully correlated with actual observed swimming competence (32). As pictorial scales have the potential to demonstrate validity as that is comparable traditional questionnaires, they are increasingly used in topics within the literature to overcome the language barriers of participants during data collection. As such, a pictorial scale may offer as a good alternative for measuring children’s HRQoL, as it may resolve the limitations of text-based parent proxy measures of HRQoL (20, 22, 29, 32).

### Development of a pictorial scale for child-HRQoL

Although there have been many developed pictorial scales that measure HRQoL for specific patients and conditions, for example, children with cochlear implants (17), the methodology of developing disease-specific scales can be different from developing a general measure of overall HRQoL, as it encompasses various aspects of an individual’s wellbeing (i.e., physical, emotional, social and functioning), whereas disease-specific scales focus on particular health conditions and their subsequent relevant symptoms/severity. As such, there is still no developed and validated HRQoL pictorial measure for children that encompasses overall components of physical health, emotional health, social health, and school functioning. Therefore, we would like to discuss and synthesize existing evidence on how researchers can effectively develop and validate a pictorial scale for overall child HRQoL. More specifically, the following synthesizes the three-phase approach of Sauer et al. (29) and Boateng and et al. (37), in addition to incorporating the Delphi method (38, 39) and Think-Aloud technique (40) to provide researchers with a comprehensive picture in understanding the process of developing and validating a pictorial scale measuring overall child HRQoL. The three-phase approach of Sauer et al. (29) was selected because they systematically reviewed 56 total existing pictorial scales and noted that there were some differences in the developmental processes. Because of these variations, Sauer et al. (29) recommended the following three-phase approach for researchers to follow when developing a pictorial scale. However, as the three-phase recommendation is only a structured guideline that does not state specific statistical techniques, we have therefore sought to incorporate a complementing scale developmental outline of Boateng et al. (37) in addition to other research methods to ensure that the pictorial scale can be developed based on a strong statistical and theoretical foundation. The following will outline and discuss synthesized 3-phase recommendations for the development of a HRQoL pictorial scale specifically.

### Development of an HRQoL pictorial scale: three-phase recommendation

#### Phase 1

The first phase of Sauer et al. (29), recommendation is to generate items by gathering ideas, understanding the construct, and brainstorm visual representations of these constructs into items. Similarly, Boateng et al. (37) suggested that researchers should consider a literature review with emphasis on HRQoL constructs and definitions of existing scales (i.e., see Child Health Questionnaire, Pediatric Quality of Life Inventory; 6, 13) and organizations (i.e., World Health Organization) as well as opinions and thoughts of experts/parents (29, 37). Researchers can either use qualitative methods/focus groups to identify parents’ perceptions of common elements reflecting HRQoL in children or include parents’/experts’ feedback in the developmental phase (17, 41). With this, age-based suitability could also be taken into account, as parents and experts may share their experience and research in relation to the child’s ability/functionality and feedback (41, 42). Researchers can also review the literature on child gross motor skills/daily living skills, primary emotions, social interactions amongst peers and school performance in creating age-appropriate scenarios (43–45).

After establishing these dimension items, common pictorial representations reflecting this can then be initially brainstormed and developed with the feedback of parents/experts. In addition to these recommendations by Sauer et al. (29), Boateng et al. (37), and Maćkiewicz and Cieciuch (22) also suggest that the developed pictorial items should represent specific situations, behaviors, and persons that are easily relatable for children during their development, allowing them easier mental representation of the item and ultimately lowering the cognitive load of the questionnaire [i.e., see Maćkiewicz and Cieciuch (22) for Pictorial Personality Traits Questionnaire example items]. Through constant feedback from parents/experts, Phase 1 ensures that the items have a clear underlying factor, are age appropriate, and are relatable in the context of children’s daily lives via multiple perspectives (29, 37).

#### Phase 2

With the initial pool of items developed in Phase 1, the second phase of Sauer et al. (29) pictorial recommendation is to conduct an interpretation check. Specifically, researchers can pre-test these questions first with parents and experts and utilize their feedback to revise the questionnaire items (if needed) prior to pilot testing it in children. The aim of these processes is to first strengthen and collect evidence for the content validity of the scale among the samples of experts, parents, and if not sufficient, utilize their comments to revise the scale. Despite the recommendations of Sauer et al. (29), no specific statistical methodology has been suggested to confirm content validity in parents/experts. However, Boateng et al. (37) suggested using formalized statistical procedures such as Cohen’s coefficient or other similar statistical methods [i.e., Aiken’s validity (46)], which can help determine statistically whether an item is able to achieve content validity.

Because Phase 2 is also a revision process, it is advised that the pictorial items be first presented to experts and parents, as they can provide valuable feedback from the child’s perspective. Moreover, pictorial items that do not reach significance or agreement by the panel should be further improved or redrawn based on feedback. The improved items should then be rerated by the panel after redevelopment until an agreement is reached. Within the ratings or discussions, it is important that parents and experts be asked to view the questionnaire from the perspective of a child and to step into their shoes to see from their perspectives in addition to giving their own opinions (22, 29). This approach can not only encourage discussions about developmental stage suitability but also allows investigation of questionnaire clarity, accuracy and reliability for children.

As the parent/expert revision process can be never-ending, researchers can also consider using the Delphi method to ensure the eventual convergence of opinions and consensus in item development. The Delphi method is a structured revision approach designed to avoid group biases such as groupthink and dominant personalities when there is a panel involved in multiple revision ratings, making it largely relevant to the current purpose (38, 39). This method involves a systematic process in which a panel (parents/experts, in this case) initially evaluates the pictorial items, and agreement can be calculated. From this, items that did not reach an agreement were revised using feedback and presented to the panel again with the accompanying agreement scores and anonymous comments of the previous round. The presentation of these materials is the core component of the Delphi method, as individual panel members can then understand other panel members’ opinions and thoughts (38, 39). Subsequent rounds of expert/parent ratings can then consider others’ thoughts before making their own ratings and comments. It is believed that this process will eventually lead to group consensus, ensuring that the pictorial items are in agreement. It is therefore suitable to recommend that researchers adopt this method in Phase 2 in addition to Sauer et al.’s (29) methodological and statistical recommendations when developing items of a pictorial questionnaire to avoid perpetual revisions.

After a successful agreement of the panel of parents and experts on the pictorial items, researchers can then pilot test the scale and establish content validity among the primary respondents, in this case, children. To make the completion and review of images easier for children, researchers can incorporate the qualitative think-aloud technique. To use this technique, researchers can first present the children with the pictorial item, while researchers can then ask the children to describe what is happening in the picture and encourage them to verbally express their thought processes, allowing researchers to understand their thoughts and perspectives (40). Through this process, researchers can then see whether the pictorial items convey the correct situation, scenario, behavior or psychological status from a child’s perspective.

Through the revision process and pilot testing in children in Phase 2, researchers can therefore revise the developed items and ensure a stronger foundation by establishing content validity before moving toward Phase 3, which aims to test other psychometric properties.

#### Phase 3

Phase 3 of Sauer et al. (29) and Boateng et al. (37) recommendation is the final phase, which assesses the psychometric properties of the pictorial scale with primary respondents, specifically convergent validity, criterion validity, discriminant validity, factorial validity, internal consistency and test–retest reliability (29). To assess the validity and reliability of scales, researchers can incorporate reference scales from established questionnaires, and when available, children’s responses on child-suited measures should be prioritized over parent-proxy questionnaires to establish psychometric properties. However, existing HRQoL reference scales are predominantly traditional questionnaires, which are susceptible to parent proxy bias or is unavailable for children under 5 years of age. It is therefore necessary not only to utilize only age-suited questionnaires (validating them to those aged 5+), but also to utilize observational/objective methods to aid in establishing different types of validities. For example, potential questions could ask how many hospital visits their children recently had and how many days their children were absent from school due to sickness, which could be an indicator of physical health, or the number of friends they made in school, which could be an indicator of social health. With the help of these observational-based questions, we can more accurately establish the convergent validity and criterion validity of the scale, as set forth by Sauer et al. (29) and Boateng et al. (37).

In terms of the statistical processes, weighing in the statistical suggestions of Boateng et al. (37) in relation to our current topic of HRQoL, convergent validity can be established when the factors of the pictorial scale are positively related to the factors of another HRQoL scale (47). Criterion validity can be established when the pictorial scale items are negatively related to health-related outcomes, such as hospital visits or days absent from school (48). Discriminant validity can be established when the pictorial scale is statistically independent from other non-HRQoL scales (49). Factorial validity can be confirmed using confirmatory factor analysis or exploratory structural equation modeling, with the fit indices showing acceptable standards. Internal consistency can be investigated by calculating the consistency of responses within the same factor structure, while test–retest reliability can be established via the similarity of answers of the same participant in a short two-timepoint study (50). All of these types of validity and reliability are important for testing and hence confirming its psychometric properties when developing a scale to ensure its strong foundation for further research and/or clinical usage.

Through the use of Sauer et al.’s (29) recommendations, Boateng et al.’s (37) recommendations, and the incorporation of the Delphi method (38, 39), the think-aloud technique (40), and observational measures, the developmental foundation for a HRQoL pictorial questionnaire could be systematically strengthened. When combined with statistical proof, the questionnaire can be a valid and reliable pictorial scale that can be a promising tool utilized by different healthcare professionals, caregivers, teachers, and researchers.

## Conclusion

HRQoL is a useful tool for quantifying one’s health satisfaction and functioning. It is particularly important to measure children’s HRQoL, as it is the prime time for growth and development. Traditional measures of HRQoL rely heavily on parent-proxy reports or age-suited questionnaires, but recent research has suggested that pictorial questionnaires can serve as an alternative tool for assessing child HRQoL. In comparison to traditional questionnaires, pictorial questionnaires are more enjoyable, demand fewer cognitive resources, and may even reach younger demographics that were previously limited. Although pictorial questionnaires can be an alternative approach that minimizes the barriers of traditional questionnaires, there has not been a pictorial questionnaire in regards to overall HRQoL. Therefore, further research is warranted to follow the recommendations of the current evidence synthesized above to develop a pictorial HRQoL questionnaire.

## Author contributions

TT: Writing – original draft. MW: Writing – review & editing. J-BL: Writing – review & editing. DC: Writing – review & editing.
